# Modelling of neocortical neural dynamics during human focal seizures

**DOI:** 10.1186/1471-2202-15-S1-P20

**Published:** 2014-07-21

**Authors:** Ernest Ho, Wilson Truccolo

**Affiliations:** 1 Department of Neuroscience, Brown University, Providence, RI, 02912, USA; 2 Center for Neurorestoration and Neurotechnology, Department of Veterans Affairs, Providence, RI, USA

## 

**Figure 1:**
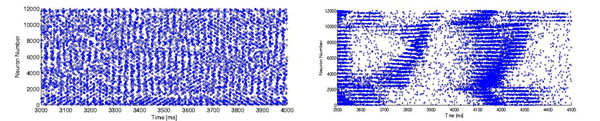
Neuronal spiking raster plots (gamma-band seizure: left; SWC seizure: right).

Recent advances in microelectrode array (MEA) recordings have made possible the simultaneous recordings of spiking activity in ensembles of neocortical single neurons during human focal seizures. As a result, a much more detailed picture of seizure initiation and spread has begun to emerge [1]. Here, we combine single neuron spiking and local field potential (LFP) data obtained from 96-MEA recordings with mathematical modelling to characterize the synaptic coupling and parameters required to sustain the neural dynamics observed in the two main types of epileptic seizures identified in the studied patients: spike-wave-complex (SWCs ~2-3 Hz) seizures and gamma-band (40-60 Hz) seizures, respectively. We model the neocortex under a 96-MEA (4mm x 4mm) with Izhikevich type neurons [2,3]. There are 28x28 minicolumns in the model. Each minicolumn is 25μm apart from each other and has 12 excitatory neurons and 4 interneurons. The neurons are synaptically connected up to a radius of 75μm and a synaptic delay of 2ms. (Connectivity drops to zero close to the MEA boundaries). Each neuron is driven by an independent Poisson input simulating background synaptic activities (i.e. high Poisson rates close to a few kHz, but individual Poisson events are of small amplitude). We simulate each of the two types of epileptic seizures by controlling the synaptic strengths amongst neurons and the Poisson drive. Dynamics similar to that in gamma-band seizures (Fig. 1) were obtained in models that combined strong mutual inhibitory synaptic couplings (g_i->i_), strong inhibitory to excitatory synaptic couplings (g_i->e_), and strong Poisson drive to both the pyramidal and interneuronal populations. The resulting single neuron spiking patterns (in both the inhibitory and excitatory cases) remained irregular, despite that they lead to sustained narrow gamma band LFP oscillations, consistent with the observations from the neuronal spiking data recorded from the patients. Dynamics similar to SWC seizures were obtained by decreasing both mutual inhibitory coupling (g_i->i_ ) and inhibitory to excitatory coupling (g_i->e_), as well as by reducing the Poisson drive to the inhibitory interneuron populations. During each SWC discharge (Figure 1), we observed the spread of neuronal spiking activity and recruitment of pyramidal cells starting from a focus, accompanied by strong coherent LFP. In the model, the participation of interneurons in SWC generation is minimal due to the weak g_i->i,e_ and low Poisson drive. Our model suggests that gamma-band seizures require strong inhibitory conductance and participation of both excitatory and inhibitory populations, while SWC seizures require weaker inhibitory conductances and are mostly driven by excitatory (pyramidal) neurons. We are working towards a conductance-based network model in which intra and extracellular ionic concentrations are included to explore how disregulation of ionic concentrations can trigger each of the two types of seizures.
